# Stability of a Tick-Borne Flavivirus in Milk

**DOI:** 10.3389/fbioe.2016.00040

**Published:** 2016-05-11

**Authors:** Danielle K. Offerdahl, Niall G. Clancy, Marshall E. Bloom

**Affiliations:** ^1^Laboratory of Virology, Rocky Mountain Laboratories, National Institute of Allergy and Infectious Diseases (NIAID), National Institutes of Health (NIH), Hamilton, MT, USA

**Keywords:** tick-borne flavivirus, goat milk, raw milk, virus stability, alimentary infection

## Abstract

The tick-borne flaviviruses (TBFV) occur worldwide and the tick-borne encephalitis virus (TBEV) members of the group often cause severe, debilitating neurological disease in humans. Although the primary route of infection is through the bite of an infected tick, alimentary infection through the consumption of TBEV-contaminated dairy products is also well-documented and is responsible for some disease in endemic areas. Experimental infection of goats, cattle, and sheep with TBEV shows that the virus can be excreted in the milk of infected animals. Additionally, the virus remains infectious after exposure to low pH levels, similar to those found in the stomach. To evaluate the survival of virus in milk, we studied the stability of the BSL-2 TBFV, Langat virus, in unpasteurized goat milk over time and after different thermal treatments. Virus was stable in milk maintained under refrigeration conditions; however, there was a marked reduction in virus titer after incubation at room temperature. High temperature, short time pasteurization protocols completely inactivated the virus. Interestingly, simulation of a typical thermal regime utilized for cheese did not completely inactivate the virus in milk. These findings stress the importance of proper milk handling and pasteurization processes in areas endemic for TBEV.

## Introduction

The tick-borne encephalitis virus (TBEV) group of tick-borne flaviviruses (TBFV) are pathogenic members of the *Flaviviridae* family. TBEV are commonly distributed across Europe and Asia and are further divided, based on geography, into three main subtypes: European, Siberian, and Far Eastern. There are two autochthonous TBFV in North America: Powassan virus and the closely related deer tick virus (Ebel, 2010). All of these viruses are associated with human disease and can cause severe, chronic, and debilitating neurological disease. An effective vaccine exists for TBEV (Lehrer and Holbrook, 2011), but even so, there are thousands of infections each year, and the incidence of TBFV infections appears to be increasing worldwide (Kunze and ISW-TBE, 2007; Piantadosi et al., 2016). The naturally attenuated Langat virus (LGTV) is employed as a convenient BSL-2 model for the more pathogenic viruses (Mitzel et al., 2007).

Tick-borne flaviviruses are most often disseminated *via* the bite of an infected tick; however, there are well-documented reports of alimentary transmission from infected, domesticated animals in endemic areas. This most often occurs through consumption of unpasteurized “raw” goat milk (Balogh et al., 2010; Caini et al., 2012) and cheese (Holzmann et al., 2009), although cow milk (Caini et al., 2012) and sheep milk (Gresikova et al., 1975) have been implicated in disease transmission. In the case of the Middle Eastern flavivirus, Alkhumra virus, an association with unpasteurized camel milk, has been suggested as a possible viral source (Alzahrani et al., 2010). Regions most often reporting alimentary transmission include Austria (Holzmann et al., 2009), Czech Republic (Aendekerk et al., 1996; Zeman et al., 2004; Kriz et al., 2009), Hungary (Balogh et al., 2010; Caini et al., 2012), Russia (Vereta et al., 1991), Slovakia (Gresikova et al., 1975; Kohl et al., 1996; Labuda et al., 2002), and Slovenia (Hudopisk et al., 2013).

Alimentary-transmitted disease, historically called biphasic milk fever, begins after a shorter incubation period (3–4 days) than that seen from a tick bite (7–14 days) (Bogovic and Strle, 2015). The initial phase of illness presents with fever, fatigue, body pains, and headache. Following an asymptomatic period of 2–10 days, the second phase involves inflammation of the central nervous system (CNS). CNS disease manifests as encephalitis, meningitis, myelitis, or as a combination of any of these. The second phase of illness can last from 3 to 21 days (Ruzek et al., 2010); however, chronic disease has been reported (Mlera et al., 2014).

The natural history of TBFV infection in domestic animals is not well studied. Goats infected subcutaneously with TBEV showed no signs of illness and no change in body temperature. However, virus could be identified by mouse inoculation and RT-PCR in milk samples that were obtained from day 2 to day 23 postinfection (Balogh et al., 2012). Similar studies with cattle (Gresikova, 1958a) and sheep (Gresikova, 1958b) also found viral shedding in the milk of experimentally infected animals. Testing of domestic animals after a suspected alimentary outbreak has shown seropositivity in goats, cows, and sheep, but milk samples are frequently negative for virus (Zeman et al., 2004; Balogh et al., 2010; Caini et al., 2012). This may be due to the length of time between diagnosis of human disease and sampling of suspected animals.

With the popularity of raw milk consumption rising, there is a potential for an increase in the number of cases of alimentary TBEV in endemic areas. Although it is clear that infected animals can produce milk containing TBFV, no recent studies have examined the stability of the virus in raw milk. In our study, we utilized the BSL-2 LGTV to model infection of raw goat milk and to demonstrate the stability of LGTV in both raw milk and laboratory cell culture medium. Additionally, we performed pasteurization and simulated cheese making with LGTV-spiked raw goat milk to determine virus stability under these conditions.

## Materials and Methods

### Cells and Viruses

Vero (African green monkey kidney) cells (ATCC) were maintained in Dulbecco’s minimal essential media [(DMEM), Life Technologies] supplemented with 50 μg/ml gentamicin and 10% fetal bovine serum (FBS) (complete DMEM) at 37°C in 5% CO_2_.

Langat virus (TP21 strain) (originally provided by Dr. Alexander Pletnev, NIH/NIAID) was prepared by infection of Vero cell cultures at a multiplicity of infection of 0.005. Virus was titrated *via* immunofocus assay, as previously described (Offerdahl et al., 2012), and stored at −80°C until use. For the immunofocus assays, the primary antibody used was mouse monoclonal anti-E 11H12 (IgG2a, a kind gift from Dr. Connie Schmaljohn, USAMRID, Fort Detrick, Frederick, MD, USA), and the secondary antibody was anti-mouse horseradish peroxidase (Dako).

### Time Course of LGTV Viability in Milk

Freshly collected, unpasteurized goat milk was obtained from a local supplier, stored at 4°C, and used the same day. LGTV was adjusted to a final concentration of 10^6^ focus forming units per milliliter (ffu/ml) in either unpasteurized goat milk or complete DMEM. Five replicate 1 ml aliquots were aliquoted into sterile screw cap tubes (Sarstedt) for each time point, media, and incubation temperature. Samples were incubated at either 4 or 22°C for 0, 8, 24, 48, or 72 h. At each time point, five replicates for each milk and DMEM were placed at −80°C and stored until viral titration was performed (as above). Serial dilutions of each replicate were made in complete DMEM (10^−1^ to 10^−5^) and tested along with the undiluted sample.

### Heat Treatment

To simulate heat pasteurization conditions (International Dairy Foods Association, 2016), LGTV was added to a final concentration of 10^6^ ffu/ml in unpasteurized goat milk or complete DMEM. Samples of the inoculated media (milk or DMEM) were aliquoted into 0.2 ml PCR tube strips (Bio-Rad, 100 μl per tube, one tube strip per replicate). A DNA engine tetrad thermocycler (Bio-Rad) was used for performing heat pasteurization of the samples. Samples were subjected to “high temperature, short time” pasteurization (HTST) by heating to 72°C for 15 s, immediately cooled to 4°C, and then placed at −80°C until viral titration was performed. To simulate the heating process involved in cheese making, samples were also slowly heated at 0.1°C/min to 30°C, then cooled to 22°C, and incubated for 16 h; samples were then placed at −80°C until viral titration. Viral titration was done by pooling eight 100 μl samples (one tube strip) per replicate.

### Statistical Analyses

Time course data were transformed into log scale using GraphPad Prism 6 (GraphPad Software, Inc.). Mean values were computed for the repetitions and two-way ANOVA performed using Sidak multiple comparisons test. Results were not considered to be statistically significant unless a *p*-value of <0.05 was observed.

## Results

### Time Course of LGTV Viability in Raw Goat Milk and DMEM

Fresh, unpasteurized goat milk was obtained from a local goat breeder and, along with complete DMEM, was added to a final concentration of 10^6^ ffu/ml LGTV TP21 in quintuplicate 1 ml aliquots. Aliquots were incubated after inoculation for 0, 8, 24, 48, or 72 h at a temperature of either 4 or 22°C. After all time points had been collected, residual virus titer was determined by immunofocus.

**Figure 1:**
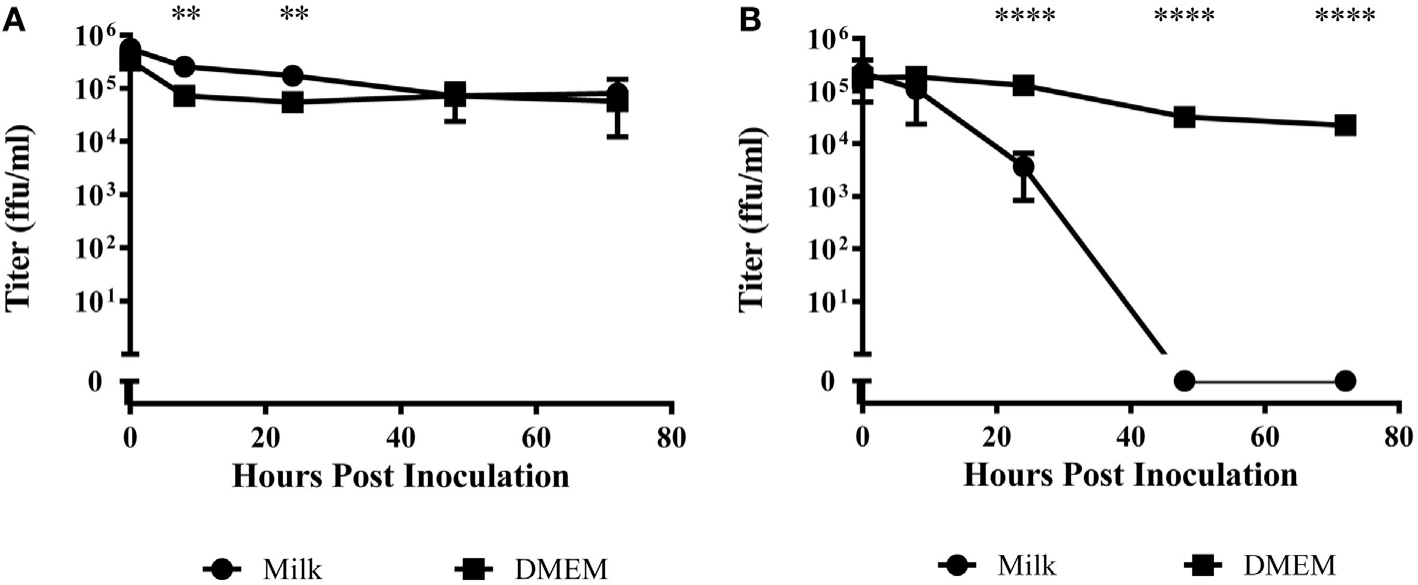
**Time course of viral titer during incubation in raw goat milk or DMEM**. Milk or DMEM was inoculated with 10^6^ focus forming units per milliliter (ffu/ml). After incubation at **(A)** 4°C or **(B)** 22°C, virus was titered in quintuplicate by immunofocus assay. Error bars represent SEM for the five replicates. Asterisks indicate statistical significance in the difference in virus titer between DMEM and goat milk (**p < 0.01; ****p < 0.0001).

When LGTV in DMEM was incubated at 4°C, there was an approximate 0.5 log_10_ decrease over the 72-h period (Figure 1A). Virus incubated in goat milk at 4°C was similarly stable, although we observed a small but significant difference at 8 and 24 h (*p* < 0.01) (Figure 1A). These findings demonstrated the stability of LGTV in goat milk held for several days at refrigerated temperatures.

At 22°C, there was a modest decrease over 72 h of nearly 2 log_10_ in LGTV in DMEM (Figure 1B). However, virus incubated in goat milk at room temperature significantly decreased by 24 h and was undetectable at time points after 48 h (*p* < 0.0001) (Figure 1B). Thus, infectious virus did not survive in goat milk held at ambient temperature.

### LGTV Viability after High Temperature, Short Time Pasteurization

A method of pasteurization termed “high temperature, short time” (HTST) is one of the most common ways that milk is pasteurized commercially for consumption (International Dairy Foods Association, 2016). HTST pasteurization specifies that milk is heated to 72°C for 15 s, and then immediately cooled to refrigeration temperatures (4°C). To test the ability of HTST pasteurization to inactivate LGTV virus, we used a thermocycler to perform HTST pasteurization on milk or DMEM samples inoculated with 10^6^ ffu/ml LGTV. No virus could be detected following this treatment, indicating that pasteurization should be effective in inactivating TBEV.

### Partial Simulation of Cheese Making and LGTV Stability

**Figure 2:**
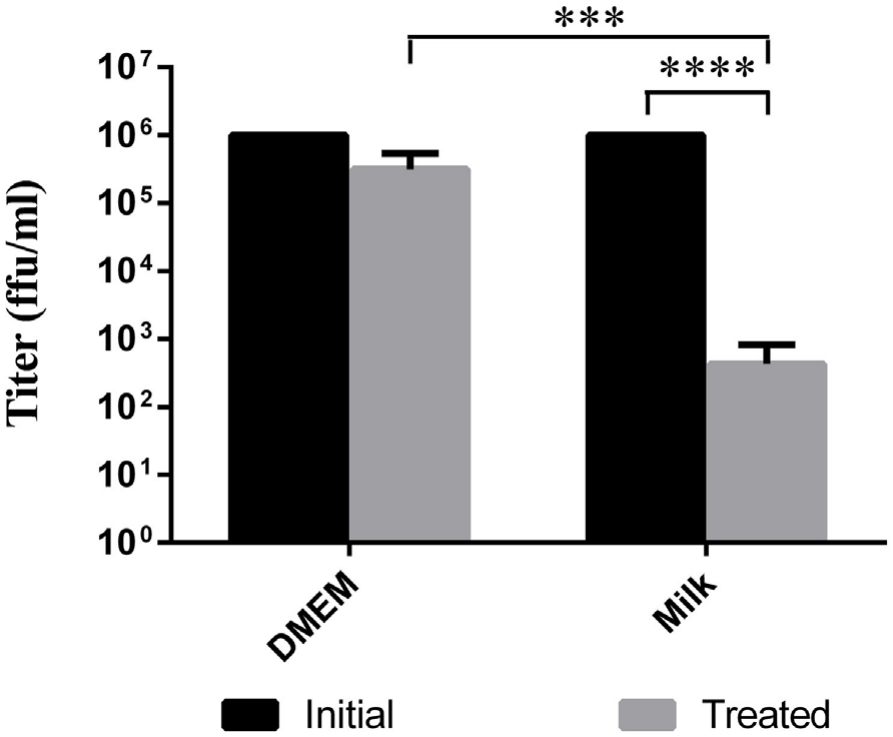
**Viability of virus after cheese making temperature treatment**. Milk or DMEM was inoculated with 10^6^ ffu/ml LGTV TP21. Samples were then slowly heated to 30°C, cooled to 22°C, and incubated for 16 h. Virus was titered by immunofocus assay. Error bars indicate SEM for five replicates. Asterisks show statistical significance in virus titer between initial inoculum and treated milk (****p < 0.0001) and between treated DMEM and treated milk (***p = 0.0002).

Some reports describe TBEV infection following consumption of cheese made with milk from infected animals (Holzmann et al., 2009). Using goat milk or cell media spiked with LGTV, we simulated the thermal segments of the cheese making process to try to evaluate the stability of the virus during this procedure. In a thermocycler, the virus containing milk or DMEM was slowly heated to 30°C. Once the milk or DMEM reached this temperature, it was allowed to gradually cool to 22°C and incubated for 16 h. This heating process reduced the viral load by a factor of about 2 log_10_ in goat milk samples compared to the initial inoculum (*p* < 0.0001) and by approximately 0.5 log_10_ in DMEM samples (Figure 2); this reduction in viral load was statistically significant when comparing treated DMEM and goat milk (*p* = 0.0002). Thus, the treatment reduced the quantity of virus, but there was still residual virus in the samples.

## Discussion

Our studies showed that the stability of TBFV in goat milk depends upon storage conditions. If the milk is held refrigerated, virus is quite stable for at least 72 h of incubation. On the contrary, if the milk is held at an ambient temperature, virus is present at 24 h but declines to undetectable levels by 48 h. Additionally, virus was completely inactivated under conditions designed to simulate high temperature, short incubation pasteurization. Furthermore, residual infectious virus was readily detected in infected milk and subjected to a temperature process designed to simulate cheese making. Thus, our experimental results demonstrated the survival of a TBFV, LGTV, in unpasteurized milk and, furthermore, suggested that alimentary transmission of TBEV in endemic areas can result from residual virus in milk and milk products.

While many developed countries require pasteurization of milk products (Food and Drug Administration, 1992; National Dairy Code, 1997; Food Standards Australia New Zealand, 2015; Food Standards Agency, 2016), the sale of unpasteurized products is allowed in some locales. Some consumers believe that raw milk tastes better or is more nutritious than pasteurized milk. Some farming families may consume raw milk for convenience. More recently, in parts of Europe and Russia, raw milk vending machines have become available, so that the public may purchase local, unprocessed milk products (Giacometti et al., 2015). Consumers of milk from vending machines are, in some cases, required to boil raw milk before consumption (Tremonte et al., 2014), but there is no enforcement of this requirement. Indeed, an Italian survey found that 43% of the consumers fail to boil vending machine milk before consumption (Giacometti et al., 2012). Recently, small outbreaks have been tied to contaminated milk consumption by families (Aendekerk et al., 1996; Kohl et al., 1996; Holzmann et al., 2009; Kriz et al., 2009) or customers of small farms (Balogh et al., 2010; Caini et al., 2012). In endemic regions, it is possible that alimentary TBEV cases may continue to increase with the growing popularity of raw milk consumption. The increase in the number of artisanal cheese producers may also prove to be a factor.

Surveys of domestic livestock within TBEV endemic regions have shown sheep, goats, and cattle to be seropositive (Juceviciene et al., 2005; Sikutova et al., 2009; Cisak et al., 2010; Klaus et al., 2012). Historically, most alimentary TBEV infections have been suspected to be from ingestion of infected goat milk products, but worldwide, cow milk and cheese are produced in larger quantity than goat or sheep milk and cheese (FAOSTAT, 2015). We have not tested cow’s milk, but it seems likely that virus could also survive in unpasteurized cow’s milk.

Milk is a worldwide dietary staple, which is composed of water, protein, fats, carbohydrates, amino acids, minerals, vitamins, and somatic cells (Quigley et al., 2013). These rich components are able to support populations of beneficial and potentially detrimental bacteria as well as fungi and viruses (Berlutti et al., 2011; Quigley et al., 2013). Milk also contains bioactive components; lactoferrin, an antimicrobial and antiviral protein (van der Strate et al., 2001; Berlutti et al., 2011), has shown ability to inhibit mosquito-borne flavivirus receptor binding (Chien et al., 2008). Our studies showed that TBFVs can survive in milk products, but it is undetermined if the virus within the milk is cell-free or cell-associated. Studies show that cell-free virus exhibits considerable stability in the presence of gastric acid (Pogodina, 1958) and across a wide range of pH conditions (Gresikova-Kohutova, 1959). Thus, it is possible that cell-free virus is the source of infectivity in alimentary infections. However, milk also contains a variety of host cells. Somatic cells found in milk are commonly cells of the innate immune system such as lymphocytes, neutrophils, and macrophages as well as mammary epithelial cells (Boutinaud and Jammes, 2002). Within cow milk, macrophages are the most common cell type, whereas neutrophils account for up to 75% of the cells in goat milk (Paape et al., 2001; Boutinaud and Jammes, 2002; Li et al., 2014). As some of these cell types are thought to be the targets for TBFV replication, it is conceivable that alimentary transmission of TBFVs results from infected cells within the milk. In addition, the secretion of goat milk is generally thought to occur *via* an apocrine process involving incorporation of ER-synthesized milk components into milk fat globules and engulfment of the globules by the apical cell membrane, resulting in retention of a small portion of the cell’s cytoplasm (cytoplasmic fragments) with the globules upon release into the lumen (Dulin et al., 1982; Mather and Keenan, 1998; Neveu et al., 2002). The source of TBFV in milk might be an interesting area of investigation, particularly, since goats do not show physical signs from TBFV infection (Balogh et al., 2012). No one has looked for the presence of TBFVs in the milk of TBFV-infected rodent models, although there are methods for milking mice (Ragueneau, 1986; Boumahrou et al., 2009).

We also tested the stability of a TBFV in cell culture media (DMEM) under various laboratory conditions. Similar to virus in milk, no significant change was seen in viral titer over the course of a 72-h incubation period under refrigeration conditions. Surprisingly, the virus was significantly more stable at room temperature and during heating to 30°C in complete DMEM supplemented with glucose and FBS than in milk. The reason for this is not immediately obvious.

In summary, our study using LGTV, a BSL-2 model for TBEV, strongly supports alimentary transmission as a valid route for TBFV infection in endemic regions. Pasteurization of milk before ingestion or further processing into dairy products was shown to prevent retention of infectious virus, while heating to 30°C fails to eliminate the risk of disease. Further studies to characterize how virus gets into the milk of infected animals might be warranted.

## Author Contributions

DO and NC performed the experiments. DO and MB designed the experiment and analyzed data. DO and MB wrote the manuscript.

## Conflict of Interest Statement

The authors declare that the research was conducted in the absence of any commercial or financial relationships that could be construed as a potential conflict of interest.
